# Endoscopic resection versus esophagectomy for T1bN0M0 esophageal cancer: a systematic review and meta-analysis

**DOI:** 10.1093/dote/doag083

**Published:** 2026-08-11

**Authors:** Nader M Hanna, Bright Huo, Zuhaib M Mir, John Agzarian

**Affiliations:** Department of Surgery, McMaster University, Hamilton, Canada; Department of Surgery, Southlake Health, Newmarket, Canada; Department of Surgery, McMaster University, Hamilton, Canada; Department of Surgery, Dalhousie University, Halifax, Canada; Department of Surgery, McMaster University, Hamilton, Canada

**Keywords:** endoscopic resection, esophageal cancer, esophagectomy, meta-analysis, systematic review

## Abstract

Esophagectomy remains the standard of care for T1b esophageal cancer. Guidelines support the use of endoscopic resection (ER) including endoscopic mucosal resection (EMR) or endoscopic submucosal dissection (ESD) in carefully selected patients. We compared patients with T1b esophageal cancer who underwent ER or esophagectomy. We searched MEDLINE, EMBASE and CENTRAL from inception to November 2025 for studies comparing esophagectomy and ER for patients with T1b esophageal cancer. We excluded studies if patients received neoadjuvant or adjuvant treatment. Outcomes were 5-year overall survival (OS) and cancer specific survival (CSS), recurrence, and mortality. Meta-analysis (with propensity-matched data when available) was performed, with a subgroup analysis of ESD versus esophagectomy. Ten retrospective studies were identified comprising 2491 patients who underwent ER (*n* = 890) or esophagectomy (*n* = 1601). Mean age was 65.7 years with 16.1% female. Six studies grouped EMR and ESD together, three reported ESD alone, one examined EMR only. OS and CSS were available in seven and four studies, respectively. There was no significant difference between esophagectomy and ER regarding 5-year OS (HR 1.26; 95% CI 1.00–1.60; *P* = 0.05), 5-year CSS (HR 0.65; 95% CI 0.20–2.17; *P* = 0.49), and 30-day mortality (HR 0.39; 95% CI 0.13–1.16; *P* = 0.09). Recurrence was 18.1% after esophagectomy and 17.7% after ER. Subgroup analysis showed no difference in OS or mortality. Risk of bias was high in five studies. Available retrospective comparative evidence suggests no clear difference between ER and esophagectomy for patients with T1b esophageal cancer. Randomized controlled trials are needed to reduce patient heterogeneity.

## INTRODUCTION

Esophagectomy remains the standard treatment for patients with T1b N0 M0 esophageal cancer based on current guidelines,^1^ yet esophagectomy for esophageal cancer is associated with a 60% complication rate.^2^ Most patients with esophageal cancer in North America are elderly and comorbid,^3^ making them at increased risk of postoperative complications. With the increase in incidence of esophageal cancer globally,^4^^,^^5^ more people will require treatment. Specifically, with increases in clinical application of advanced endoscopic surveillance with esophageal ultrasonography, there is an increase in patients being diagnosed with T1b N0 esophageal cancer.^6^

Endoscopic resection for early-stage esophageal cancer, with either endoscopic mucosal resection (EMR) or endoscopic submucosal dissection (ESD), is becoming more prevalent^7^ and has been integrated into recent guidelines.^8^^,^^9^ This organ-preserving procedure is often performed on an outpatient basis, can be done without general anesthesia, has a much lower complication rate, can be offered to patients who may not tolerate an esophagectomy,^10^ and is potentially curative. Despite these advantages of endoscopic resection over esophagectomy, lymph node harvesting is not possible with endoscopic resection; therefore, esophagectomy allows for better prognostication and thus a higher likelihood of adjuvant treatment if warranted.^11^

Several studies have attempted to compare outcomes of patients with T1b N0 esophageal cancer who undergo endoscopic resection or esophagectomy but have resulted in conflicting conclusions regarding recurrence and survival.^7^^,^^12–14^ We therefore performed a systematic review and meta-analysis of the current literature. We aimed to compare oncological outcomes between endoscopic resection and esophagectomy for patients with T1bN0M0 esophageal cancer, with a subgroup analysis that compares ESD with esophagectomy.

## METHODS

### Search strategy

A systematic review and meta-analysis were undertaken and reported in accordance with the Preferred Reporting Items for Systematic Reviews and Meta-Analyses guidelines.^15^ We queried the Ovid Medline, Embase, and SCOPUS databases from inception to November 2025 for articles written in English and performed on human subjects. Gray literature was searched without restriction. Search terms included ‘esophageal cancer,’ ‘esophagectomy,’ ‘endoscopic submucosal dissection’, ‘endoscopic mucosal resection’, ‘survival’ and others, outlined in the full search strategy found in Appendix 1.

### Study selection

We included articles on patients with T1b esophageal cancer that were treated with either endoscopic resection or with esophagectomy. There was no restriction on the type of esophagectomy. All comparative study designs were included. Studies which included patients who underwent esophagectomy after an endoscopic resection were excluded. Studies which included patients receiving previous treatment for esophageal cancer before the intervention or comparator were excluded. Published systematic reviews, society guidelines, and position papers were hand-searched for articles not identified in the initial search, then subsequently excluded from the pooled meta-analysis.

### Outcomes assessed

The primary outcome of this study was 5-year overall survival, chosen because it is the most reported outcome in the literature and is a widely used metric to describe survival in esophageal cancer. Secondary outcomes include 5-year cancer specific survival, recurrence, postoperative complication rate, and mortality.

### Data extraction and risk of bias assessment

Two reviewers independently screened abstracts and then full text reviews using the Covidence^16^ platform which has been shown to be superior to other software for de-duplication.^17^ Discrepancies were resolved by a senior author. Data extraction was then performed on the eligible articles using the same software by two independent reviewers, with the senior reviewer available to resolve discrepancies. All included studies were assessed for the risk of bias using either the Cochrane Risk of Bias 2.0 tool (for RCTs) or the Newcastle Ottawa Scale tool (for observational studies). Two reviewers independently rated each study. Discrepancies between reviewer ratings were resolved by discussion between the two reviewers or a third-party tiebreaker when necessary.

### Data analysis

When both crude and propensity-matched estimates were available, propensity-matched estimates were preferentially included in pooled analyses. Descriptive statistics were used to describe the cohort’s patient and disease characteristics. Meta-analyses were performed using JASP for all analysable outcomes. A pairwise meta-analysis was used with a random effects model to calculate risk ratios for survival. Forest plots were created for outcomes reported by three or more studies. We quantified heterogeneity between studies using the I^2^ statistic and assessed statistical significance using the Chi^2^ test. A subgroup analysis of those receiving ESD was performed.

## RESULTS

**Fig. 1:**
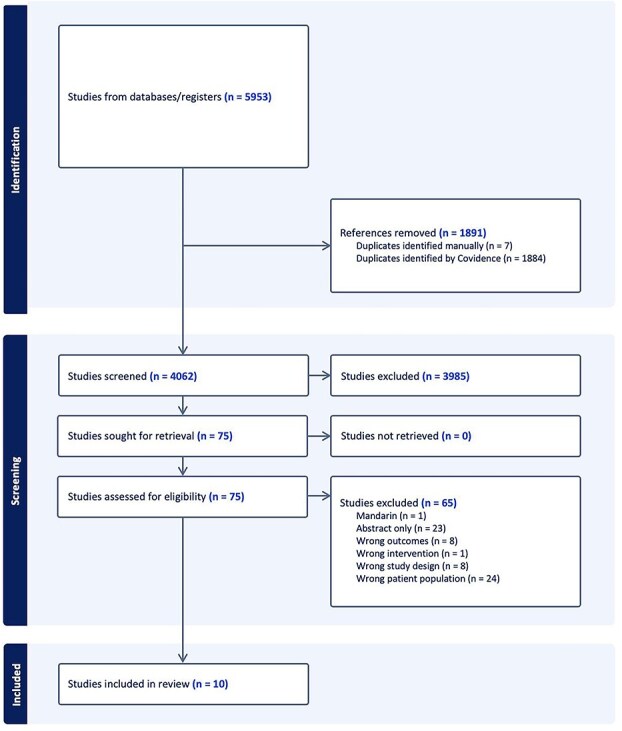
PRISMA flowsheet for included studies.

The original search revealed 5953 articles with 1891 duplicates. We screened a total of 4068 individual abstracts, and 75 full texts, which resulted in 10 articles^12–14^^,^^18–24^ for inclusion (Fig. 1).

### Study characteristics

All 10 included papers were retrospective cohort studies with five being population-level studies using healthcare databases (Table 1). Six of 10 studies were from Japan or China, three from USA, one from the UK. Two were published in 2013, the remainder were from 2018 onwards. Six of 10 studies grouped patients who underwent either EMR or ESD together in one cohort,^13^^,^^14^^,^^18^^,^^20^^,^^23^^,^^24^ three compared ESD to esophagectomy,^12^^,^^19^^,^^21^ and one compared EMR to esophagectomy.^22^ CSS and OS were reported in four^12^^,^^13^^,^^23^^,^^24^ and seven^12–14^^,^^19^^,^^20^^,^^23^^,^^24^ studies respectively. Three studies stratified patients by differentiation,^14^^,^^20^^,^^22^ and five reported lymphovascular invasion.^14^^,^^19–22^ Three studies evaluated squamous cell carcinoma (SCC) cohorts,^12^^,^^18^^,^^19^ four evaluated adenocarcinoma (AC) cohorts,^14^^,^^20^^,^^22^^,^^23^ and the remaining studies included mixed histology.^13^^,^^21^^,^^24^

**Table 1:** Study and patient characteristics of the 10 included studies

Study	Country	Study Design	Outcomes assessed	T category definition	Histology	Treatment arm	N	% female	Age, years (SD/range)	Differentiation	LVI
Yamauchi 2021	Japan	Single center	OS CSS	Pathological	SCC	ESD	12	-	-	-	-
						Esophagectomy	13	-	-	-	-
Wang 2021	China	SEER	OS CSS	Not mentioned	Mix	EMR + ESD	70	-	-	-	-
						Esophagectomy	69	-	-	-	-
Kamarajah 2022	UK	NCDB	OS Mortality	Clinical	AC	EMR + ESD	329	14.3	-	Moderate 193 Poor 136	46
						Esophagectomy	329	16.7	-	Moderate 204 Poor 125	55
Akutsu 2013	Japan	Single center	Recurrence	Pathological	SCC	EMR + ESD	15	-	67.2 (8.6)	-	-
						Esophagectomy	89	-	62.4 (8.0)	-	-
Ngamruengphong 2013	USA	SEER	OS CSS	Clinical	AC	EMR + ESD	39	-	71 (11)	-	-
						Esophagectomy	523	-	63 (9)	-	-
Nelson 2018	USA	Single center	Recurrence	Clinical	AC	EMR	23	13.0	71.5 (63–76)	Moderate 16 Poor 7	6
						Esophagectomy	42	14.3	64.5 (61–70)	Moderate 20 Poor 22	0
Qin 2019	China	SEER	OS CSS	Clinical	Mix	EMR + ESD	43	-	-	-	-
						Esophagectomy	94	-	-	-	-
Swanson 2023	USA	NCDB	OS	Clinical	AC	EMR + ESD	256	14.5	-	Well 54 Moderate 137 Poor 65	40
						Esophagectomy	256	12.9	-	Well 57 Moderate 129 Poor 70	41
Liu 2023	China	Single center	Mortality Recurrence	Pathological	Mix	ESD	32	34.4	63.5 (8.9)	-	2
						Esophagectomy	119	26.1	61.6 (8.8)	-	18
An 2020	China	Single center	OS Mortality	Pathological	SCC	ESD	71	-	67.6 (7.7)	-	3
						Esophagectomy	67	-	66.2 (6.8)	-	3

*Abbreviations:*AC = Adenocarcinoma; CSS = Cancer Specific Survival; EMR = Endoscopic Mucosal Resection; ESD = Endoscopic Submucosal Dissection; LVI = Lymphovascular Invasion; NCDB = National Cancer Database; OS = Overall Survival; SCC = Squamous Cell Carcinoma; SD = Standard Deviation; SEER = Surveillance, Epidemiology, and End Results.

### Patient characteristics

There were 2491 patients in total across the 10 studies. Patients underwent EMR/ESD (*n* = 890) or esophagectomy (*n* = 1601). Five studies reported age^18^^,^^19^^,^^21–23^: the mean age of these 1020 patients was 65.7 years. Four studies reported sex^14^^,^^20^^,^^22^: 16.1% of these 874 patients were female. Of the three studies that reported differentiation, the EMR/ESD group contained 400 patients with well/moderate differentiation, and 208 patients with poor differentiation, whilst the esophagectomy group contained 410 and 217 patients, respectively. A total of 97 patients in the EMR/ESD group and 117 patients in the esophagectomy group had LVI across the five studies that reported this.

### 5-Year OS

**Fig. 2:**
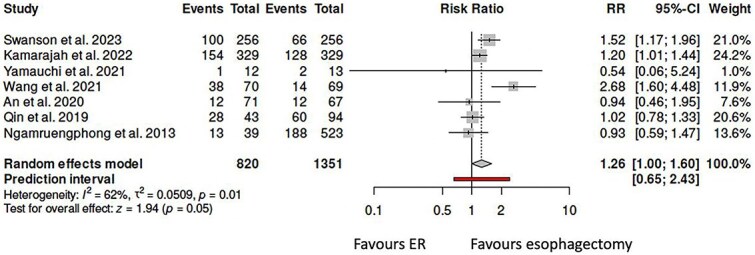
Forest plot for 5-year overall survival.

Overall survival was reported in seven studies, with five^13^^,^^14^^,^^20^^,^^23^^,^^24^ investigating EMR + ESD and two^12^^,^^19^ investigating ESD only. Pooled estimates demonstrate no statistically significant difference between esophagectomy and EMR/ESD (HR 1.26; 95% CI 1.00–1.60; *P* = 0.05; *I^2^ =* 62%) (Fig. 2), although, this borders the threshold of statistical significance favoring esophagectomy.

### 5-Year CSS

**Fig. 3:**
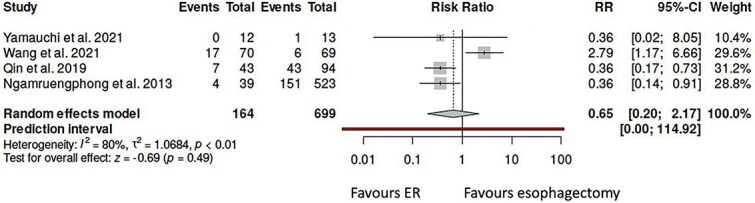
Forest plot for 5-year cancer specific survival.

Cancer specific survival was available from four studies.^12^^,^^13^^,^^23^^,^^24^ There was no statistically significant difference between esophagectomy and EMR/ESD from the pooled data (HR 0.65; 95% CI 0.20–2.17; *P* = 0.49; *I^2^ =* 80%) (Fig. 3).

### Recurrence

Recurrence was evaluated in three studies^18^^,^^21^^,^^22^ and reported as either lymph node or hematological recurrence,^18^ any recurrence,^21^ or local versus regional versus distal recurrence.^22^ To increase statistical power, we grouped all recurrences together. Our pooled data demonstrated a total recurrence rate of 18.1% in the EMR/ESD group and 17.7% in the esophagectomy group (*P* = 0.94).

### 30-Day mortality

**Fig. 4:**
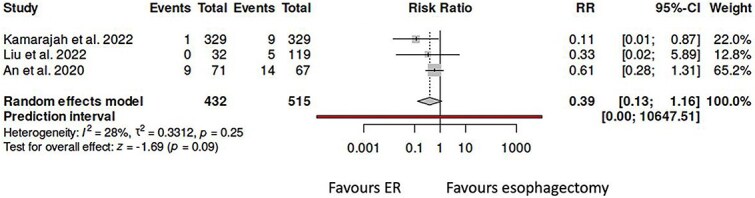
Forest plot for 30-day mortality.

30-day mortality was available from three studies.^19–21^ There was no statistically significant difference between esophagectomy and EMR/ESD from the pooled data: (HR 0.39; 95% CI 0.13–1.16; *P* = 0.09; *I^2^ = 28*%) (Fig. 4).

### Subgroup analysis

**Fig. 5:**
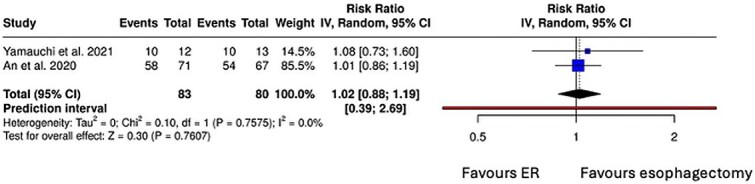
Subgroup analysis Forest plot for 5-year overall survival.

**Fig. 6:**
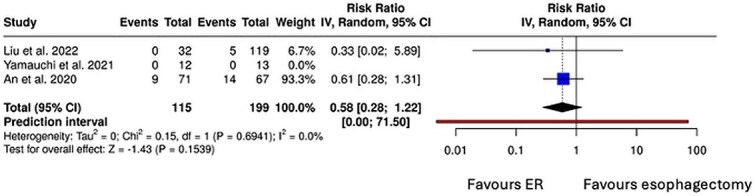
Subgroup analysis Forest plot for 30-day mortality.

Three studies compared ESD with esophagectomy,^12^^,^^19^^,^^21^ two of which reported OS.^12^^,^^19^ There was no difference in 5-year OS (HR 1.02; 95% CI 0.88–1.19; *P* = 0.76; *I^2^ =* 0%) or 30-day mortality (HR 0.58; 95% CI 0.28–1.22; *P* = 0.15; *I^2^ =* 0%) (Figs. 5 and 6, respectively). We were unable to investigate 5-year CSS in this subgroup analysis due to insufficient observations.

### Risk of bias assessments

Of the 10 studies, five were considered to have a high risk of bias^14^^,^^18^^,^^21–23^ and five had a low risk of bias.^12^^,^^13^^,^^19^^,^^20^^,^^24^ Of those judged to have a high risk of bias, four of these were based on comparability.^14^^,^^18^^,^^22^^,^^23^ One was deemed to have a high risk of bias in all domains^18^ (Supplementary File).

## DISCUSSION

Esophagectomy remains standard of care for most T1b esophageal cancer patients but is associated with a high morbidity and mortality.^2^ EMR/ESD can be a reasonable option for some patients with T1b esophageal cancer who would otherwise not be suitable for esophagectomy. Our systematic review and meta-analysis compared cancer outcomes in patients with T1b esophageal cancer who underwent either esophagectomy (without neoadjuvant or adjuvant therapy) or EMR/ESD across 10 retrospective cohort studies. Our study demonstrates no statistically significant difference in 5-year overall survival, 5-year cancer specific survival, or 30-day mortality between those that underwent esophagectomy alone or EMR/ESD alone. This finding was consistent in a subgroup analysis comparing ESD and esophagectomy.

Although not reaching statistical significance (*P* = 0.05), there was a trend toward improved 5-year OS in those that underwent esophagectomy (HR 1.26) versus EMR/ESD. Chen et al also did not identify a statistically significant difference in overall survival (HR 1.02, *P =* 0.94) in their systematic review, but they compared T1b esophageal cancer patients who had esophagectomy to those who received EMR/ESD and adjuvant chemoradiotherapy. It is possible that in our study, patients who were older, more comorbid, and more frail were offered EMR/ESD, therefore died from any cause sooner than their healthier counterparts who underwent esophagectomy. Five-year CSS was also not statistically different between both groups in our study (HR 0.65; 95% CI 0.20–2.17; *P* = 0.49), however did err on the side of being worse in the EMR/ESD group. Overall survival should be interpreted with caution, as patients undergoing EMR/ESD were likely more frail than surgical candidates in these retrospective studies. In contrast, cancer-specific survival and recurrence outcomes may more accurately reflect oncologic effectiveness, although these outcomes remain subject to residual confounding. The lack of statistical significance seen in OS and CSS may in part be due to the heterogenous nature of our pooled cohort, impacted by selection bias within the individual studies. Our cohort included patients with SCC and AC, SM1–3, EMR and ESD, and clinical and pathological stage, representing a very heterogenous cohort of patients with T1b esophageal cancer. It is possible that a more homogenous group of patients may have resulted in a statistically significant difference in favor of esophagectomy in both OS and CSS. Zheng et al^25^ also performed a systematic review and meta-analysis on T1b patients comparing endoscopic resection with esophagectomy. However, they included patients that had adjuvant therapy post esophagectomy, (which was a strict exclusion criterion in our study) which may explain their finding of better OS in the esophagectomy group.

In the included studies, not all patients were selected based on clinical T category (cT1b). In five studies, patients were included based on pathological T1b (pT1b) from the surgical or endoscopic specimen. Those that used the clinical T-category included Kamarajah^20^ (NCDB), Swanson^14^ (NCDB), Qin^24^ (SEER), Ngamruenphong^23^ (S,EER), and Nelson^22^ (pre-procedure endoscopic ultrasound). Wang^13^ also queried the SEER database but did not specify whether the T-category was clinical or pathological. The method used to identify the T-category is a critical component in cancer staging, especially in early-stage esophageal cancer where it dictates treatment choice. Most guidelines advocate for CT scan to differentiate between T1–4; however, it is less precise in delineating between cT1a and cT1b. In the preoperative phase, this differentiation is more accurately achieved with endoscopic ultrasound (EUS). Despite this recommendation, previous studies^26^^,^^27^ have showed that EUS had only a modest concordance with T category compared to pathology specimens from EMR or surgery, and that this concordance improves with higher T category.^28^ EUS has also been shown to be inconsistent regarding lymph node involvement.^29^ The accuracy of EUS is also user dependent^30^ and may have implications regarding the most optimal method of cancer staging and therefore appropriate patient selection. However, if endoscopic resection is used as a diagnostic tool rather than therapeutic, then these limitations become less relevant.

There remains a concern however, that the lack of lymphadenectomy with endoscopic resection compared to esophagectomy may result in a poorer survival.^11^ Surprisingly, we found no difference in pooled recurrence rate between those who underwent EMR/ESD and those who had an esophagectomy. Proponents of esophagectomy for T1b esophageal cancer cite the high incidence of nodal involvement at diagnosis, hence a need for thorough lymphadenectomy.^11^ Without lymphadenectomy, cancer already in the lymph nodes may further metastasize. Additionally, appropriate nodal harvest will increase accuracy of pathological staging, thus those upstaged patients who require adjuvant therapy would be more likely to receive it. As such, one would expect recurrence of any type to be higher with endoscopic resection than with esophagectomy. Another systematic review and meta-analysis^31^ reported a lower recurrence rate with esophagectomy compared to EMR/ESD for T1b esophageal cancer, though in contrast patients in the endoscopic resection group received adjuvant chemotherapy. The overlap in recurrence rates in our study may be in part attributed to the heterogenous manner in which those individual studies captured and reported their data, as such it is difficult to draw firm conclusions for this particular outcome. A randomized clinical trial comparing patients with T1b esophageal cancer who undergo esophagectomy or endoscopic resection, stratified by whether they received EMR or ESD, with SM1/SM2/SM3 subgroup analyses would provide much needed clarity on which T1b patients can receive endoscopic resection.

Another systematic review^32^ included both T1a and T1b esophageal cancers and concluded that T1b cancer should be treated with esophagectomy after demonstrating a higher lymph node positivity in patients with lymphovascular invasion and with submucosal invasion. Guidelines^1^^,^^33^ state that ESD is acceptable and appropriate for SM1 but not SM2 or SM3. Sensitivity analysis based on depth of invasion (SM category) was not possible given that this was not explicitly reported or sub-stratified in the included trials, which is a key determinant of nodal metastatic risk and treatment selection. Pooling all T1b lesions may obscure clinically meaningful differences between lower-risk SM1 tumors and higher-risk SM2/SM3 disease, potentially biasing the pooled estimates toward null findings. Nodal metastases vary widely by depth and histology, with frequencies quoted as 6%, 23%, and 58% in SM1–3 AC, respectively, and 27%, 36%, and 55% in SM1–3 SCC, respectively.^34^ Accordingly, these results should not be interpreted as evidence of therapeutic equivalence across all T1b subgroups. Additionally, that group did neither compare survival nor recurrence between both groups but did note that recurrence was 0%–17% in those who underwent endoscopic resection, which is similar to our pooled rate of 18.1%, and further commented that recurrence with ESD was lower than with EMR. Our subgroup analysis of only ESD versus esophagectomy did not demonstrate a statistically significant difference in 30-day mortality, or 5-year OS, however it is worth noting that one of the three included articles had no observations in either group.

An additional source of heterogeneity is the inclusion of both SCC and AC within the pooled cohort. These histologic subtypes differ in tumor biology, typical anatomic location, patterns of lymphatic spread, and guideline-based management, all of which may influence both recurrence and survival outcomes. Because many included studies reported mixed histology cohorts without stratified outcome reporting, a formal subgroup analysis by histology was not feasible. Accordingly, the present findings should not be assumed to apply equally to SCC and AC, and future prospective studies should report outcomes separately by histologic subtype.

There are some limitations of our study. Firstly, many of the included studies did not separate patients who had EMR from those who had ESD, thus our subgroup analysis was restricted to just three studies which resulted in only two outcomes being analysed. Further, this limits the ability to draw conclusions regarding endoscopic resection as a unified strategy. Secondly, not all included studies reported differentiation, which might serve as an uncontrolled confounding factor. Similarly, most included studies did not discriminate between SM1, SM2, or SM3, which is a critical factor in determining suitability for endoscopic therapy. There was also a mix of SCC and AC, making it difficult to provide advice on the most ideal patient to recommend for ER. Lastly, 5 of the 10 included studies were judged to have a high risk of bias, and thus these results should be interpreted with caution.

In summary, our systematic review and meta-analysis of 10 studies comprising 2491 patients from retrospective comparative data does not demonstrate a clear survival difference between EMR/ESD and esophagectomy. Endoscopic resection may serve as a potential option in carefully selected patients, particularly those who are not surgical candidates. The specific subgroup of patient and tumor biology that will benefit from endoscopic resection without an increased risk of recurrence remains uncertain and further investigation in this area is warranted.

## Supplementary Material

Supplementary_materials_doag083

## Notes

Specific author contributions: Nader M Hanna (Conceptualization, Data curation, Investigation, Methodology, Project administration, Resources, Supervision, Visualization, Writing—original draft, Writing—review & editing), Bright Huo (Conceptualization, Data curation, Methodology, Resources, Writing—review & editing), Zuhaib M Mir (Formal analysis, Investigation, Methodology, Resources, Software, Writing—review & editing), John Agzarian (Conceptualization, Supervision, Writing—review & editing).

Financial support: There was no source of funding.
