# Supplementary material for: Endoscopic resection versus esophagectomy for T1bN0M0 esophageal cancer: a systematic review and meta-analysis
Source: Dis Esophagus. 2026 Aug 11;39(4):doag083. doi: 10.1093/dote/doag083 (PMC13461216; doi:10.1093/dote/doag083)
Supplement: Supplementary file 1 [file supplementary_materials_doag083.zip › Supplementary_materials_doag083.docx]

**Search strategy**

(((Esophageal Neoplasm) OR(Neoplasm, Esophageal)OR( Esophagus Neoplasm)OR( Esophagus Neoplasms)OR(Neoplasm, Esophagus)OR (Neoplasms, Esophagus)OR (Neoplasms, Esophageal) OR(Cancer of Esophagus)OR (Cancer of the Esophagus)OR (Esophagus Cancer)OR (Cancer, Esophagus)OR (Cancers, Esophagus) OR(Esophagus Cancers) OR(Esophageal Cancer)OR (Cancer, Esophageal) OR(Cancers, Esophageal)OR (Esophageal Cancers)) AND (((((T1) OR (T1-SM)) OR (T1b-SM)) OR (T1b)))) AND (((endoscopic resection) OR (ER)) OR ((Endoscopic submucosal dissection) OR (ESD)) AND ((Surgery) OR (Surgical treatment) OR (esophagectomy))).
