# Supplementary material for: Endoscopic resection versus esophagectomy for T1bN0M0 esophageal cancer: a systematic review and meta-analysis
Source: Dis Esophagus. 2026 Aug 11;39(4):doag083. doi: 10.1093/dote/doag083 (PMC13461216; doi:10.1093/dote/doag083)
Supplement: Supplementary file 1 [file supplementary_materials_doag083.zip › T1b - ROB.docx]

**Risk of Bias Assessment of the 10 included studies**
